# Anthocyanin-Rich Fraction from Kum Akha Black Rice Attenuates NLRP3 Inflammasome-Driven Lung Inflammation In Vitro and In Vivo

**DOI:** 10.3390/nu17071186

**Published:** 2025-03-28

**Authors:** Sonthaya Umsumarng, Warathit Semmarath, Punnida Arjsri, Kamonwan Srisawad, Intranee Intanil, Sansanee Jamjod, Chanakan Prom-u-thai, Pornngarm Dejkriengkraikul

**Affiliations:** 1 Faculty of Veterinary Medicine, Chiang Mai University, Chiang Mai 50100, Thailand; sonthaya.u@cmu.ac.th; 2 Lanna Rice Research Center, Chiang Mai University, Chiang Mai 50200, Thailand; sansanee.cm@gmail.com (S.J.); chanakan.p@cmu.ac.th (C.P.-u.-t.); 3 Akkhraratchakumari Veterinary College, Walailak University, Nakhon Si Thammarat 80160, Thailand; warathit.se@wu.ac.th; 4 Centre for One Health, Walailak University, Nakhon Si Thammarat 80160, Thailand; 5 Department of Biochemistry, Faculty of Medicine, Chiang Mai University, Chiang Mai 50200, Thailand; punnida_dream@hotmail.com (P.A.); k.srisawad@gmail.com (K.S.); intranee.in@cmu.ac.th (I.I.); 6 Anticarcinogenesis and Apoptosis Research Cluster, Faculty of Medicine, Chiang Mai University, Chiang Mai 50200, Thailand; 7 Department of Plant and Soil Sciences, Faculty of Agriculture, Chiang Mai University, Chiang Mai 50200, Thailand

**Keywords:** black rice extract, anthocyanins, functional food, anti-inflammation, NLRP3 inflammasome, respiratory tract inflammation

## Abstract

Background/Objectives: Chronic lower respiratory tract inflammation can result from exposure to bacterial particles, leading to the activation of the NLRP3 inflammasome pathway. These effects may cause irreversible respiratory damage, contributing to persistent lung injury and chronic obstructive pulmonary disease (COPD), as observed in long COVID or bacterial pneumonia in older adults’ patients. Given its profound impact, the NLRP3 inflammasome has emerged as a key therapeutic target for mitigating aberrant inflammatory responses. Methods: In this study, we investigated the anti-inflammatory effects of Kum Akha black rice, a functional food, on the attenuation of NLRP3 inflammasome pathway using lipopolysaccharide-induced A549 lung epithelial cells and a C57BL/6NJcl mouse model. The anthocyanin-rich fraction from Kum Akha black rice germ and bran extract (KA1-P1) was obtained using a solvent-partitioned extraction technique. Results: KA1-P1 exhibited a high anthocyanin content (74.63 ± 1.66 mg/g extract) as determined by the pH differential method. The HPLC analysis revealed cyanidin-3-O-glucoside (C3G: 45.58 ± 0.48 mg/g extract) and peonidin-3-O-glucoside (P3G: 6.92 ± 0.29 mg/g extract) as its anthocyanin’s active compounds. Additionally, KA1-P1 demonstrated strong antioxidant activity, as assessed by DPPH and ABTS assays. KA1-P1 (12.5–100 μg/mL) possessed inhibitory effects on LPS + ATP-induced A549 lung cells inflammation through the significant suppressions of *NLRP3, IL-6, IL-1β*, and *IL-18* mRNA levels and the inhibition of cytokine secretions in a dose-dependent manner (*p* < 0.05). Mechanistic analysis revealed that KA1-P1 downregulated key proteins in the NLRP3 inflammasome pathway (NLRP3, ASC, pro-caspase-1, and cleaved-caspase-1). Furthermore, in vivo studies demonstrated that KA1-P1 significantly diminished LPS-induced lower respiratory inflammation in C57BL/6NJcl mice, as evidenced by the reduced bronchoalveolar lavage fluid and blood levels of inflammatory cytokines (IL-6, IL-1β, and IL-18) and diminished histopathological inflammatory lung lesions. Conclusions: Overall, our findings suggest that the anti-inflammatory properties of KA1-P1 may support its application as a functional supplement or promote the consumption of pigmented rice among the elderly to mitigate chronic lower respiratory tract inflammation mediated by the NLRP3 inflammasome pathway.

## 1. Introduction

Diseases affecting the lower respiratory system, particularly lung diseases, are among the leading causes of morbidity and mortality worldwide. These include bacterial and viral pneumonia, chronic obstructive pulmonary disease (COPD), and tuberculosis [1,2]. Infectious pneumonia, characterized by lung parenchyma infection caused by bacterial or viral pathogens, can overwhelm the host’s defense mechanisms. In aging populations, several factors contribute to compromised immune defense, making elderly individuals not only more susceptible to infections, but also prone to severe disease progression, prolonged recovery, and poor clinical outcomes [3,4].

Notably, pneumonia in older adults is often exacerbated by dysregulated inflammatory responses, leading to heightened lung inflammation and a significantly increased mortality rate compared to younger populations [5]. Studies have shown that innate immune cells, such as macrophages, exhibit hyperresponsiveness in aging individuals, resulting in an exaggerated inflammatory reaction. If left unchecked, this excessive immune response can lead to long-term consequences, including irreversible lung damage, acute respiratory distress syndrome (ARDS), COPD, and the persistent respiratory symptoms observed in long COVID patients [6,7]. Additionally, remaining antigens from infectious agents can remain in the blood circulation or within respiratory tissues, sustaining a state of sub-chronic and chronic inflammation [8,9]. Given these challenges, the development of therapeutic or prevention strategies targeting chronic lung inflammation, particularly in disease disorders such as acute respiratory distress syndrome (ARDS), bacterial pneumonia-induced hyperinflammation, COVID-19-associated lung injury, and age-related respiratory inflammation, is of great interest.

Despite advances in medical research, effective treatment options for chronic respiratory diseases remain limited, partly due to an incomplete understanding of disease pathogenesis and the inability to effectively suppress chronic inflammatory processes [10]. The NLRP3 inflammasome is a key mediator of innate immune activation and plays a crucial role in inflammation-driven lung diseases. Activation of the NLRP3 inflammasome can lead to irreversible structural damage in lung tissues, impairing normal pulmonary function [11]. Previous study demonstrated that mice deficient in the inflammasome adaptor protein ASC (ASC-/- mice) do not develop pulmonary fibrosis when exposed to fibrosis-inducing agents such as bleomycin, highlighting the inflammasome’s critical role in disease progression [12]. Additionally, in elderly individuals, bacterial or viral infections often trigger severe lung inflammation through NLRP3 inflammasome activation, contributing to increased mortality rates compared to younger populations [13,14]. Therefore, targeting the NLRP3 inflammasome pathway represents a promising approach for reducing lung inflammation and mitigating lung injury progression.

In Thailand and other Asian countries, rice is a staple food, with pigmented rice varieties receiving increasing attention due to their high content of bioactive compounds, including anthocyanins, proanthocyanidins, and oryzanol. These compounds exhibit diverse biological properties, including antioxidant and anti-inflammatory effects [15,16]. The germ and bran fractions of pigmented rice, particularly black and red rice, are rich in phytochemicals, with anthocyanins and proanthocyanidins being the primary bioactive constituents known for their strong antioxidant and anti-inflammation activities [17,18].

Domestic black rice varieties cultivated in northern Thailand exhibit variations in their nutrient composition, making them a potential source of functional food with enhanced health benefits. Black rice is particularly rich in anthocyanins, a class of flavonoids responsible for its characteristic purple-to-reddish pigmentation. To date, over 500 anthocyanin compounds have been identified, with cyanidin-3-O-glucoside (C3G) and peonidin-3-O-glucoside (P3G) being the predominant anthocyanins in black rice germ and bran [19,20,21]. Kum Akha 1 CMU (KA1) is a glutinous black rice variety cultivated in northern Thailand and selectively bred by the Lanna Rice Research Center, Chiang Mai University, to enhance its bioactive compound content. This variety has been reported to contain the highest levels of total phenolic compounds, total anthocyanins, and cyanidin-3-O-glucoside (C3G) among local black rice varieties [18,22,23]. Previous studies have demonstrated that C3G and P3G have anti-inflammatory potential. Specifically, black rice germ and bran extracts containing these anthocyanins were shown to inhibit inflammation induced by the SARS-CoV2-Spike S1 glycoprotein in A549 lung cells and THP-1 macrophages by downregulating NLRP3, IL-1β, and IL-18 inflammatory gene expression and suppressing the secretion of pro-inflammatory IL-6, IL-1β, and IL-18 cytokine secretions [24]. Additionally, C3G from black rice exhibited anti-inflammatory effects in lipopolysaccharide-induced RAW 264.7 macrophages and in carrageenan inflammatory induction in BALB/c mice, through the reduction in TNF-α and IL-1β secretion [25,26]. Moreover, black rice germ and bran drinking supplement, rich in total anthocyanins, improved physical performance and modulate inflammatory markers (IL-6, CRP) and IGF-1 levels in aging populations when combined with an exercise intervention [27]. Despite these favorable findings, no study has comprehensively investigated the in vitro and in vivo effects of anthocyanins from black rice as a therapeutic or preventive strategy against chronic respiratory inflammation mediated by NLRP3 inflammasome activation.

This study aimed to evaluate the anti-inflammation effects of the anthocyanin-rich fraction from KA1 black rice germ and bran extracts on LPS-induced lower respiratory inflammation in A549 lung epithelial cells (in vitro) and C57BL/6NJcl mice (in vivo). The findings from this study could provide valuable insights into the potential development of functional food supplements or dietary strategies incorporating pigmented rice to mitigate chronic lung inflammation, particularly among elderly individuals at risk for pneumonia and inflammasome-mediated respiratory diseases.

## 2. Materials and Methods

### 2.1. Chemical and Reagents

The HPLC grade of butanol, ethanol, methanol, and water solvents were supplied from RCL Labscan Limited (Bangkok, Thailand). Dulbecco’s Modified Eagle Medium (DMEM) (cat. no. 12800-017), 10x trypsin enzyme (cat. no. 15090-046), 100x penicillin-streptomycin antibiotics (cat. no. 15140-122), and fetal bovine serum (FBS) (cat. no. A5256701) from Gibco BRL (Grand Island, NY, USA) were utilized. The QIAzol lysis reagent (cat. no. 79306) was supplied from Qiagen (Valencia, CA, USA). ReverTra Ace^®^ qPCR Master Mix (cat. no. FSQ-201) was supplied from Toyobo Co., Ltd. (Osaka, Japan). SensiFAST^TM^ SYBR^®^ Lo-ROX Kit (cat. no. BIO-94005) was supplied from Meridian Bioscience^®^ (Cincinnati, OH, USA). The anti-NLRP3, anti-ASC, anti-caspase-1, and primary antibody, along with horseradish peroxidase-conjugated goat anti-mouse (cat. no. 7076S) or rabbit (cat. no. 7074S) IgG, were supplied from Cell Signaling Technology (Danvers, MA, USA). The adenosine triphosphate (cat. no. A6419), lipopolysaccharide (cat. no. L4516), and anti-β-actin primary antibody (cat. no. A5316) were supplied from Sigma-Aldrich (St. Louis, MO, USA).

### 2.2. Herb Resources and Extraction Technique

The black rice (*Oryza sativa* L.) variety Kum Akha 1 CMU, a glutinous rice, was obtained from the Lanna Rice Research Center, Chiang Mai University, Thailand. The rice was cultivated in sandy loam soil (Sansai series) during the wet season (June–November period of 2023). The rice samples were processed into unpolished rice (caryopsis) by de-husking the paddy using a rice testing machine (model P-1, Ngek Seng Huat Company, Bangkok, Thailand).

The solvent-partitioned extraction method followed a previously described protocol [28]. Black rice germ and bran were separated using a rice milling machine (Kinetic (Hubei) Energy Equipment Engineering Co., Ltd., Wuhan, China), then soaked in 50% ethanol for 48 h. The extract was filtered and concentrated using a rotary vacuum evaporator (BUCHI, Flawil, Switzerland), followed by partitioning with saturated butanol. The medium polar fraction (KA1-P1), and residual water fraction (KA1-P2) were collected, evaporated, and freeze-dried to obtain the anthocyanin-rich fraction, and stored at −20 °C for further experiments.

### 2.3. Determination of Phenolics and Flavonoids

Total phenolic content was determined using the modified Folin-Ciocalteu assay [29], with absorbance measured at 765 nm and expressed as mg gallic acid equivalents (GAE) per gram of extract (mg GA/g extract). Total flavonoid content was assessed using the aluminum chloride (AlCl_3_) colorimetric assay [30]. with absorbance recorded at 510 nm and expressed as mg catechin equivalents per gram of extract (mg catechin/g extract).

### 2.4. Determination of Anthocyanins

The amount of anthocyanins contained in the extracts were measured using the pH differential method, as previously described [31]. Briefly, the extracts were suspended in 0.1% HCl in 80% methanol and the reaction was developed for 12 h. After that, the two dilutions of the extract solutions were prepared. Briefly, the first sample (0.25 mL) was diluted with 1 mL of 0.025 M KCl buffer pH = 1.0, and the second was diluted with 1 mL of 0.45 M CH_3_COONa buffer pH = 4.5. The samples were incubated for 15 min at room temperature. At the end of incubation time, the OD absorbance of the sample was measured at 520 and 700 nm wavelengths by a UV–visible spectrophotometry instrument. The total anthocyanin content was then calculated using the following formula, where A = (A520 nm–A700 nm) pH 1.0 − (A520 nm − A700 nm) pH 4.5, MW = 449.2 g/mol (cyanidin-3-glucoside), DF = dilution factor, *ε* = molar extinction coefficient (L × mol^−1^ × cm^−1^), and L = cell path length (1 cm)

(1) $$Total\;anthocyanin\;content=\frac{A\times Mw\times DF\times 10^{3}}{\varepsilon \times L}$$

### 2.5. Identification of Cyanidin-3-O-Glucoside (C3G) and Peonidin-3-O-Glucoside (P3G) Anthocyanin Compounds in KA1 Using High-Performance Liquid Chromatography (HPLC)

High-performance liquid chromatography (HPLC) was used to identify cyanidin-3-O-glucoside (C3G) and peonidin-3-O-glucoside (P3G) in KA1 extracts, following a previously described protocol [32]. The analysis was performed using an Agilent Infinity 1260 HPLC system (Santa Clara, CA, USA) with a Zorbax Eclipse Plus C18 column (250 mm × 4.6 mm, 5 µm). Mobile phases included 0.4% trifluoroacetic acid in distilled water (A) and 0.45% trifluoroacetic acid (TFA) in acetonitrile (B) under isocratic conditions. The detection wavelength was 520 nm, with a flow rate of 1.0 mL/min for 30 min. The temperature of the column was set at 40 °C. Injection volumes were 10 μL, with sample concentrations of 5000 μg/mL and 10,000 μg/mL, and C3G and P3G standard concentrations at the concentration of 0–100 μg/mL.

### 2.6. Determination of Antioxidant Properties by ABTS and DPPH Assays

ABTS and DPPH assays were performed to evaluate the antioxidant properties of KA1-P1 and KA1-P2, as has been previously described [33,34]. For the ABTS assay, the absorbance was measured at 734 nm using a spectrophotometer and compared to a calibration curve of Trolox, which served as the positive control. For the DPPH assay, 20 μL of various concentrations of KA1-P1 or KA1-P2 were mixed with 180 μL of freshly prepared DPPH methanolic solution and incubated in the dark for 10 min. The absorbance was then recorded at 540 nm and compared to a calibration curve of Vitamin E, which was used as the positive control. The antioxidant capacity of the extracts was expressed as the half-maximal inhibitory concentration (IC_50_).

### 2.7. Cell Cultures

The A549 lung epithelial cell line was obtained from the American Type Culture Collection (ATCC). In normal monolayer culture, A549 cells exhibit a polygonal shape and a sheet-like growth pattern, consistent with their epithelial origin [35]. Cells were maintained as a monolayer in DMEM supplemented with 10% FBS, 2 mM L-glutamine, 50 U/mL of penicillin, and 50 μg/mL of streptomycin. Cultures were incubated in a humidified atmosphere of 5% CO_2_ at 37 °C. Once the cells reached 70–80% confluency, they were harvested and used for subsequent experiments.

### 2.8. Determination of Cytotoxicity of KA1 Extracts

The cytotoxicity of KA1-P1 and KA1-P2 against A549 lung cells was assessed using the 3-(4,5-dimethylthiazol-2-yl)-2,5-diphenyltetrazolium bromide (MTT) assay. Briefly, A549 cells were seeded at a density of 3 × 10^3^ cells per well in culture medium. Once the cells formed a monolayer and reached 70–80% confluency, they were treated with varying concentrations of KA1-P1 or KA1-P2 (ranging from 0 to 200 μg/mL) for 24 and 48 h. After the incubation period, 10 μL of 0.5 mg/mL MTT solution in phosphate-buffered saline (PBS) was added to the cells, and they were incubated for an additional 4 h. The culture supernatant was then carefully discarded, and the cells were resuspended in 200 μL of DMSO to dissolve the MTT formazan crystals. Absorbance was measured at 540 and 630 nm using a UV–visible spectrophotometry instrument (BioTek Synergy H1, Winooski, VT, USA). The assay was conducted in triplicate for each concentration of KA1 extract. Cell viability was determined by comparing the absorbance values to those of the control group and expressed as a percentage relative to the control.

### 2.9. Determination of the Inhibitory Effects of KA Extract on Inflammatory Cytokine Secretions

The secretion levels of IL-6, IL-1β, and IL-18 in the culture medium of A549 cells were assessed using an enzyme-linked immunosorbent assay (ELISA) kit (BioLegend, San Diego, CA, USA), following the manufacturer’s protocol [24]. Briefly, A549 cells (3 × 10^5^ cells/well) were seeded in a 6-well culture plate and allowed to adhere overnight. Once the cells formed a monolayer and reached 70–80% confluency, they were pretreated with varying concentrations (0–100 μg/mL) of KA1-P1 for 24 h. The cells were then stimulated with 1 μg/mL lipopolysaccharide (LPS) for 6 h, followed by treatment with 5 nM adenosine triphosphate (ATP) for 30 min to induce an inflammatory response. After the incubation period, the culture medium from each well was collected for ELISA analysis. Absorbance was measured at 450 and 570 nm using a microplate reader (Sunrise, Tecan Trading AG, Männedorf, Switzerland). Cytokine secretion levels were determined in triplicate and quantified by comparison with standard calibration curves for each cytokine.

### 2.10. Determination of the Inhibitory Effects of KA Extract on Inflammatory Gene Expressions

To determine the inflammatory gene expressions, A549 cells (3 × 10^5^ cells/well) were seeded in a 6-well plate and allowed to adhere overnight. When the cells created a monolayer and reached 70–80% confluency, the A549 cells were pretreated with KA1-P1 (0–100 μg/mL) for 24 h and then exposed to 1 μg/mL LPS for 6 h and 5 nM ATP for 30 min, respectively.

To assess the expression of inflammatory genes, A549 cells (3 × 10^5^ cells/well) were seeded in a 6-well plate and incubated overnight for adherence. Once the cells reached 70–80% confluency, they were pretreated with KA1-P1 (0–100 μg/mL) for 24 h, followed by stimulation with 1 μg/mL LPS for 6 h and 5 nM ATP for 30 min. Total RNA was extracted from treated cells using Qiazol reagent, and its concentration and purity were determined using a NanoDrop 2000/2000c spectrophotometer (Thermo Fisher Scientific, Waltham, MA, USA) to ensure suitability for further analysis. Reverse transcription was performed using a Mastercycler^®^ nexus gradient machine (Eppendorf, GA, Hamburg, Germany), and quantitative real-time PCR was conducted using the qRT-PCR ABITM 7500 Fast and 7500 real-time PCR machine (Thermo Fisher Scientific, Waltham, MA, USA). Gene expression levels were analyzed using the QuantStudio 6 Flex real-time PCR software v1.0 (Applied Biosystems, Waltham, MA, USA), and the results were calculated using the 2^−ΔΔCT^ method, normalized to GAPDH and control samples.

The primer sequences for IL-6 were obtained from Bio Basic Canada Inc. (Markham, ON, Canada), while those for NLRP3), IL-1β, IL-18, and GAPDH were sourced from Humanizing Genomics Macrogen (Geumcheongu, Seoul, Republic of Korea). All primer sequences used in this study are listed in Table 1.

### 2.11. Determination of the Inhibitory Effects of KA1 Extract on NLRP3 Inflammasome-Associated Proteins Expressions

To examine the effect of KA1-P1 on NLRP3 inflammasome machinery proteins in LPS and ATP-induced lung cell inflammation, protein expression levels were evaluated using Western blotting analysis. A549 cells (3 × 10^5^ cells/well) were seeded in a 6-well culture plate and allowed to adhere overnight. Once the cells reached 70–80% confluency, they were treated with KA1-P1 (0–100 μg/mL) for 24 h, followed by stimulation with 1 μg/mL LPS for 6 h and 5 nM ATP for 30 min. After treatment, cells were harvested and lysed using RIPA buffer, and protein concentration was determined using the Bradford assay (Thermo Scientific, Waltham, MA, USA). Whole-cell lysates were subjected to 12% SDS-PAGE for protein separation and subsequently transferred onto nitrocellulose membranes. The membranes were blocked with 5% BSA in 0.1% TBS-Tween and washed twice with 0.1% TBS-Tween. They were then incubated overnight at 4 °C with primary antibodies against NLRP3 (1:1000), ASC (1:2000), or caspase-1 (1:1000). Following incubation, the membranes were washed 5 times with 0.1% TBS-Tween and further incubated with horseradish peroxidase-conjugated anti-mouse or rabbit IgG (1:10,000), depending on the primary antibody, for 2 h at room temperature. After another five washes with 0.1% the TBS-Tween, bound antibodies were detected using a chemiluminescent detection system (Cytiva, Marlborough, MA, USA) and then exposed to the iBright™ CL-1500 imaging system (Thermo Fisher Scientific, Waltham, MA, USA). To ensure equal protein loading, membranes were stripped and re-probed with an anti-β-actin antibody (1:10,000). Band intensity was analyzed using ImageJ 1.410 software.

### 2.12. Animal Model

Six to eight-week-old C57BL/6NJcl mice (*n* = 40, male and female, weight = 28–30 g) were supplied from Nomura Siam International, Bangkok, Thailand. The mice were acclimatized to the animal laboratory facility for at least 7 days. The male and female animals were housed separately in a clean environment and handled according to the animal laboratory institutional guidance. All animals were maintained under a constant 12 h/12 h light–dark cycle. The humidity levels were maintained at 50 ± 10%, at a temperature of 21 ± 1 °C. All mice were provided with free access to food and water.

### 2.13. In Vivo Study of the Anti-Inflammatory Effects of KA1-P1 on LPS-Induced Respiratory Inflammation

To evaluate the anti-inflammatory activity of KA1-P1, blood-based inflammatory cytokines and histopathological lung lesions were assessed. In this study, inflammation was induced in a mouse model using a modified protocol previously described [37,38]. Experimental mice were nebulized with 3 mg/mL of LPS in 0.9% normal saline solution (NSS), while the negative control group received 0.9% NSS without LPS.

Briefly, C57BL/6NJcl mice (*n* = 40), were randomly divided into five groups (*n* = 8 per group, with an equal number of males and females, no inclusion and exclusion criteria were set) by an independent researcher. The sample size was calculated based on G*power (version 3.19.4), with a confidence interval of 95%. Except for the normal control group, all mice were exposed to 3 mg/mL of LPS in 0.9% NSS via nebulization (three times, every three days) in a plastic container. Mice were then treated with either sterilized distilled water (negative control group), KA1-P1 at a low dose (1000 mg/kg) or high dose (2000 mg/kg), or prednisolone (1 mg/kg, positive control group). Treatments were administered daily via oral gavage for 12 weeks. The normal control group received sterilized distilled water (DW) without LPS induction and continued receiving sterile DW daily for 12 weeks.

No physically detectable adverse effects were observed in any mice throughout the experiment. At the end of the experiment (week 12), whole blood was collected, and the mice were sacrificed using isoflurane gas in a sealed chamber. Mice were placed into an oxygen case and filled with overdosing levels of isoflurane anesthetic agent vapor as a standard euthanasia protocol [39]. Internal organs, including the kidneys, heart, spleen, liver, and lungs, were harvested. Whole lung lavage was performed using pre-warmed 0.9% NSS to obtain bronchoalveolar lavage fluid (BALF). Lung tissues were fixed for histological analysis in a 10% formalin buffer solution, embedded in paraffin, sectioned at 4 μm thickness, and stained with hematoxylin and eosin (H&E). The stained sections were examined under a digital whole-slide scanner (Pannoramic MIDI II, 3DHISTECH Ltd., Budapest, Hungary) and photographed at 10× magnification. The in vivo anti-inflammatory effects of KA1-P1 were evaluated by measuring blood-based inflammatory cytokines (IL-6, IL-1β, and IL-18) and assessing histopathological lung lesions. Data analysis was performed by a separate researcher who was unaware of the group’s identities until the final statistical comparisons were made.

The animal experimental design was approved by the Ethics Committee of Laboratory Animal Unit, Faculty of Medicine, Chiang Mai University (Protocol code MC008/2567[01/2567-02-05,], date of approval; August 2024). All methods were performed following the relevant guidelines and regulations. This study follows the recommendation in the ARRIVE guidelines.

### 2.14. Statistical Analysis

All data were presented as mean ($\bar{X}$) ± standard deviation (S.D.) values or standard error (S.E.) values. Statistical analysis was analyzed using Prism version 8.0 software. The statistical testing was determined using the independent *t*-test or one-way ANOVA with Tukey’s test. The statistical significance was determined at * *p* < 0.05, ** *p* < 0.01, and *** *p* < 0.001.

## 3. Results

### 3.1. Phytochemical Characteristics of Kum Akha 1 (KA1) Black Rice Germ and Bran Extracts

Two fractions of KA1 extracts, KA1-P1 and KA1-P2, were obtained using the solvent-partitioned extraction technique. The percentage yields of KA1-P1 and KA1-P2 were 5.21 ± 0.38% (*w*/*w*) and 7.36 ± 0.25%, respectively. The phytochemical characteristics of KA1-P1 and KA1-P2 are shown in Table 2. The results indicate that KA1-P1 contained significantly higher levels of total phenolics, total flavonoids, and total anthocyanins compared to KA1-P2 (*p* < 0.05). Specifically, the total anthocyanin content in KA1-P1 was 74.63 ± 1.66 mg/g extract.

**Figure 1:**
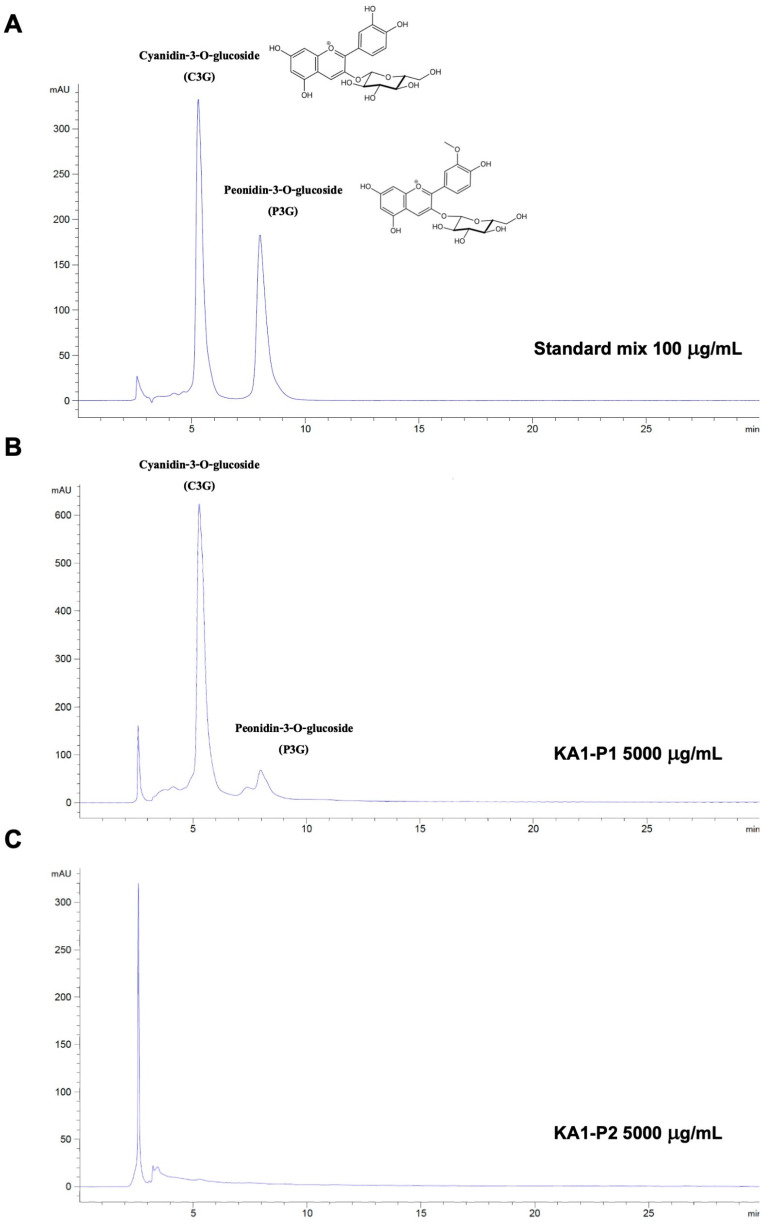
HPLC profile of Kum Akha 1 (KA1) black rice germ and bran extract. Chromatograms of anthocyanin standards (C3G and P3G) at 100 μg/mL (**A**), KA1-P1 extract at 5000 μg/mL (**B**), and KA1-P2 extract at 5000 μg/mL (**C**) were analyzed using a reversed-phase C18 column. The mobile phase consisted of 0.4% trifluoroacetic acid in water (**A**) and 0.45% trifluoroacetic acid in acetonitrile (**B**) under isocratic conditions. Detection was set at 520 nm at a flow rate of 1.0 mL/min.

Black rice contains mainly two anthocyanin compounds, C3G and P3G [15,18], as shown in the HPLC chromatogram, where the standards used in this study are presented in Figure 1. Quantification of anthocyanins in KA1-P1 and KA1-P2 based on the HPLC chromatogram revealed that KA1-P1 contained high levels of both C3G (45.58 ± 0.48 mg/g extract) and P3G (6.92 ± 0.29 mg/g extract), whereas these two anthocyanins were not detected in KA1-P2. These findings suggest that C3G and P3G are the predominant anthocyanins present in KA1-P1 and serve as the primary bioactive compounds in the anthocyanin-rich fraction, contributing to the observed anti-inflammatory effects.

### 3.2. Antioxidant Capacity of KA1 Black Rice Germ and Bran Extracts

The free radical-scavenging properties of KA1 extracts were evaluated using DPPH and ABTS assays. As shown in Table 3, the inhibitory concentration (IC_50_) of the positive control for DPPH assay, vitamin E, was 24.74 ± 0.60 µg/mL, while the IC_50_ of the positive control for ABTS assay, Trolox, was 2.45 ± 0.23 µg/mL. KA1-P1 exhibited significantly stronger antioxidant activity than KA1-P2, as indicated by its lower IC_50_ values in both DPPH and ABTS tests. Based on its phytochemical composition and antioxidant capacity, KA1-P1 was selected for further investigation of its biological activity in inhibiting inflammatory responses in A549 lung epithelial cells and in an animal model.

### 3.3. Cell Viability Effects of KA1-P1 on A549 Lung Epithelial Cells

**Figure 2:**
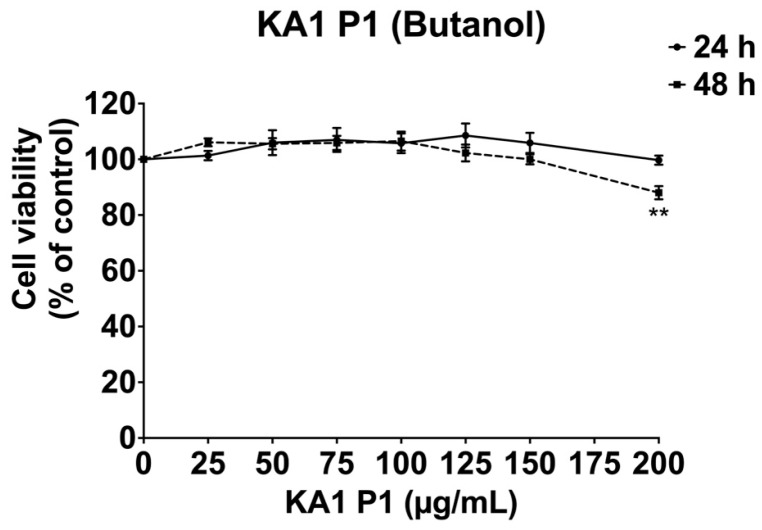
Cytotoxicity assessment of KA1 black rice germ and bran extract on A549 lung cells. A549 cells were exposed to variable concentrations of KA1-P1 ranging from 25 to 200 μg/mL for 24 h and 48 h. The cell viability was determined using the MTT assay. The presented data represent the mean ± S.D. values obtained from at least in triplicate experiments. ** *p* < 0.01 indicate statistically significant differences compared with the cell viability of the control (0 μg/mL) for the respective incubation time.

Before assessing the anti-inflammatory properties of KA1-P1, we first evaluated its potential cytotoxic effects on A549 cells. The MTT assay results are presented as the percentage of control cell viability following incubation with increasing concentration of KA1-P1 (0–200 μg/mL), as shown in Figure 2. The findings revealed that KA1-P1 did not exhibit cytotoxicity within this concentration range after 24 h and 48 h of treatment. Based on these results, KA1-P1 was deemed non-toxic to A549 cells and was therefore suitable for further experimentation.

### 3.4. Inhibitory Effects of KA1-P1 on Inflammatory Cytokine Secretions in LPS and ATP-Induced A549 Lung Cells

**Figure 3:**
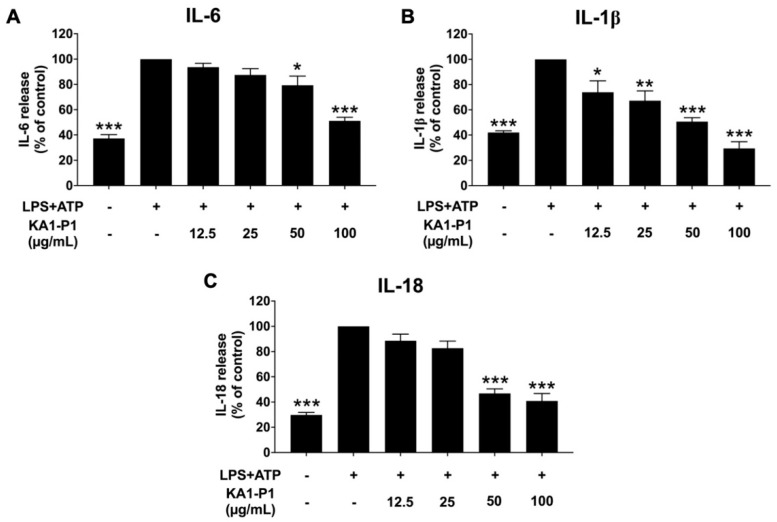
Inhibitory effects of KA1-P1 on inflammatory cytokine secretions in LPS + ATP-induced A549 cells. The cells were pre-treated with KA1-P1 (12.5–100 μg/mL) for 24 h before LPS (1 μg/mL, 6 h) and ATP (50 nM, 30 min) exposure. Cytokine levels of IL−6 (**A**), IL−1β (**B**), and IL−18 (**C**) were measured via ELISA. The LPS + ATP−induced A549 cells are presented as 100% of the control. Data are mean ± S.D. from at least in triplicate experiments; * *p* < 0.05, ** *p* < 0.01, and *** *p* < 0.001 indicate statistically significant differences compared with the LPS + ATP−induced control group.

To evaluate the anti-inflammatory effects of KA1-P1 on respiratory tract inflammation, we first determined its inhibitory effects on cytokine release in LPS + ATP-induced A549 lung cells. Following LPS + ATP stimulation, the release of pro-inflammatory cytokines, including IL-6, IL-1β, and IL-18, was measured using ELISA, as shown in Figure 3. The results demonstrated that LPS + ATP stimulation significantly increased the release levels of IL-6, IL-1β, and IL-18 compared to the non-LPS + ATP control group (*p* < 0.001) indicating a strong inflammatory response in A549 cells. However, pretreatment with KA1-P1 at varying concentrations (12.5–100 μg/mL) for 24 h significantly decreased the release of IL-6, IL-1β, and IL-18 in a dose-dependent manner (*p* < 0.05). These findings suggest that KA1-P1 effectively suppresses LPS + ATP-induced inflammation in A549 cells.

### 3.5. Effect of KA1-P1 on the Inhibition of IL-6, IL-1β, IL-18, and NLRP3 Gene Expressions in LPS + ATP-Exposed A549 Cells

**Figure 4:**
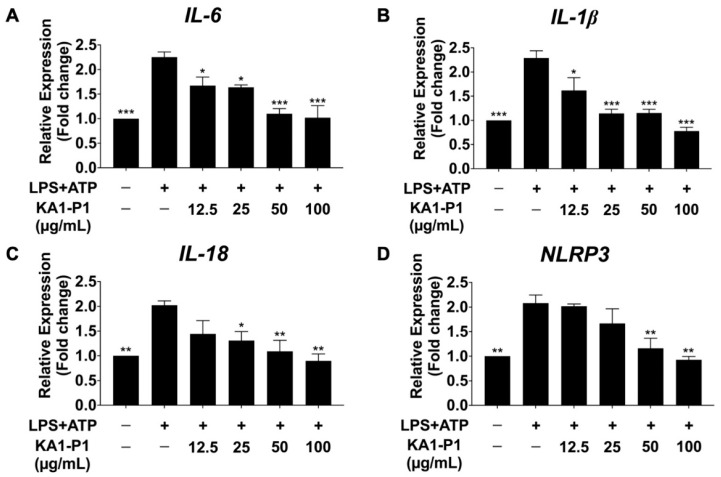
Inhibitory effects of KA1-P1 on IL-6, IL-1β, IL-18, and NLRP3 mRNA expressions in LPS + ATP-induced A549 cells. The cells were pre-treated with KA1-P1 (12.5–100 μg/mL) for 24 h before LPS (1 μg/mL, 6 h) and ATP (50 nM, 30 min) exposure. The mRNA expressions of IL-6 (**A**), IL-1β (**B**), IL-18 (**C**), and NLRP3 (**D**) were assessed using RT-qPCR. The LPS + ATP-induced A549 cells are presented as 100% of the control. Data are mean ± S.D. from at least in triplicate experiments; * *p* < 0.05, ** *p* < 0.01, and *** *p* < 0.001 indicate statistically significant differences compared to the LPS + ATP-induced A549 cells.

To further investigate the in vitro anti-inflammatory effects of KA1-P1 on respiratory tract inflammation, we examined its impact on inflammatory responses at transcriptional level by assessing the expression of inflammatory-related genes, including, IL-6, IL-1β, IL-18, and NLRP3. The mRNA expression levels in LPS + ATP-induced A549 lung cells were determined by qRT-PCR, as shown in Figure 4. The results indicated that IL-6, IL-1β, and IL-18, and NLRP3 expression levels were significantly upregulated in LPS + ATP-treated cells compared to the non-LPS + ATP control group (*p* < 0.01), confirming that an inflammatory response was induced at the transcriptional level. However, pretreatment with KA1-P1 at varying concentrations (12.5–100 μg/mL) for 24 h significantly downregulated IL-6, IL-1β, and IL-18 and NLRP3 mRNA expression in a dose-dependent manner (*p* < 0.01). These findings suggest that KA1-P1 effectively attenuates LPS + ATP-induced inflammatory responses in A549 cells at both the gene and protein levels.

### 3.6. Inhibitory Effects of KA1-P1 on the NLRP3 Inflammasome Pathway in LPS + ATP-Induced A549 Cells

The activation of inflammatory cytokine genes and the subsequent release of cytokines, particularly IL-1β and IL-18, are partially regulated by the upstream NLRP3 inflammasome pathway. The NLRP3 inflammasome complex consists of NLRP3, ASC, and pro-caspase-1, which is activate into cleaved caspase-1. Activation of the inflammasome pathway facilitates the interaction between NLRP3 and ASC, leading to the recruitment of pro-caspase-1 and its conversion into cleaved caspase-1. This process subsequently upregulates inflammatory gene expression, promoting the synthesis and release of IL-1β and IL-18 cytokines [14,40].

**Figure 5:**
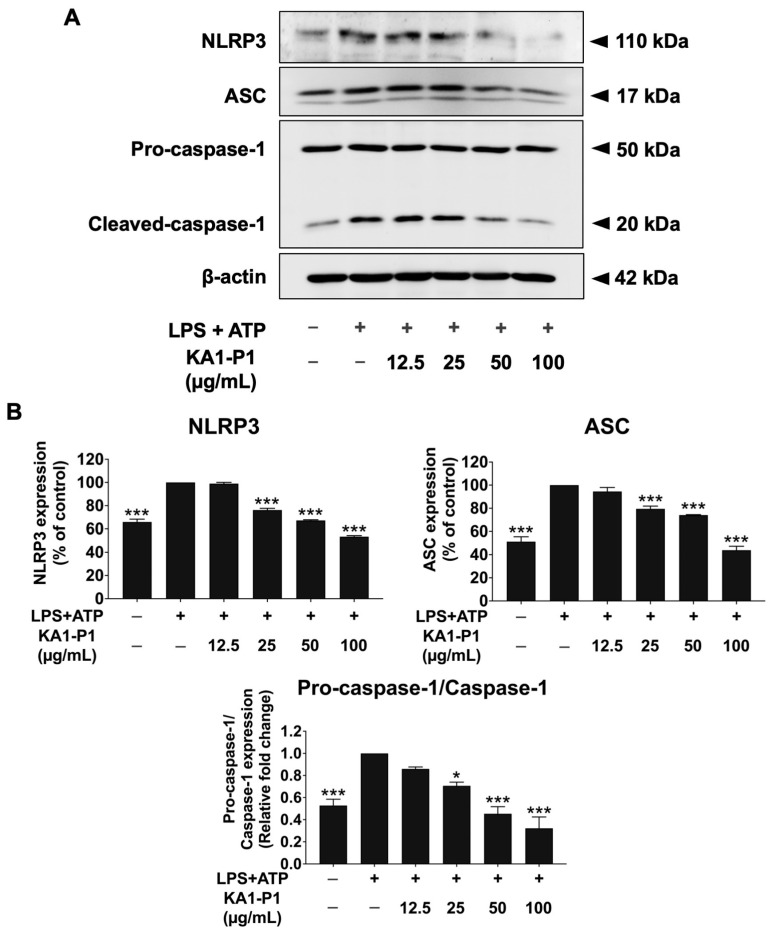
Mechanistic study on the attenuation of NLRP3 inflammasome pathway in LPS + ATP-induced A549 lung cells by KA1-P1. The cells were pre-treated with KA1-P1 (12.5–100 μg/mL) for 24 h before LPS (1 μg/mL, 6 h) and ATP (50 nM, 30 min) exposure. The data were visualized as bands via Western blotting analysis (**A**), and the band density measurements (**B**) were performed. The LPS + ATP-induced A549 cells are presented as 100% of the control. Data are mean ± S.D. from at least triplicate experiments; * *p* < 0.05 and *** *p* < 0.001 indicate statistically significant differences compared to the LPS + ATP-induced A549 cells.

To determine whether the anti-inflammatory effects of KA1-P1 were mediated through the inhibition of the NLRP3 inflammasome pathway, we analyzed the expression of NLRP3 inflammasome-related proteins in LPS + ATP-induced A549 cells using Western blotting, as shown in Figure 5. The results demonstrated that the expression levels of NLRP3, ASC, pro-caspase-1, and cleaved caspase-1 were significantly upregulated in LPS + ATP-treated cells compared to the non-LPS + ATP control group (*p* < 0.01). However, KA1-P1 treatment significantly downregulated the expression of these inflammasome-related proteins in a dose-dependent manner (*p* < 0.001). Overall, these findings suggest that the inhibitory effects of KA1-P1 on the in vitro respiratory inflammation in LPS + ATP-induced A549 cells were, at least in part, due to the suppression of NLRP3 inflammasome protein expression, leading to reduced pro-inflammatory cytokine gene expression and cytokine release.

### 3.7. In Vivo Inhibitory Effects of KA1-P1 on Lower Respiratory Inflammation in LPS + ATP-Induced C57BL/6NJcl Mice

**Figure 6:**
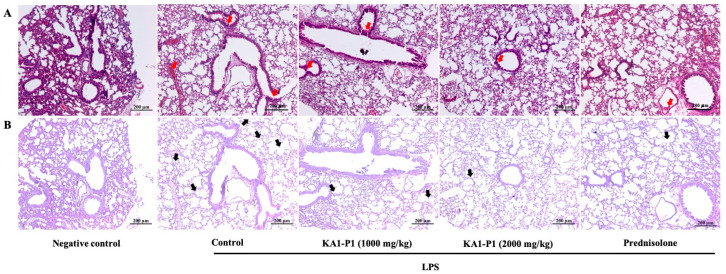
Hematoxylin and eosin staining of lung tissues (**A**) demonstrates the presence of peribronchiolar inflammation (red arrow), and periodic acid–Schiff (PAS) stain of lung tissues (**B**) demonstrates the presence of alveolar macrophages (black arrow) present in the C57BL/6NJcl mice The black scale bars represent 200 µm.

To evaluate the anti-inflammatory properties of KA1-P1 in vivo, an LPS-induced respiratory inflammation model was utilized in C57BL/6NJcl mice, as described in the methodology. Both the high- and low-dose KA1-P1 treatments obviously attenuated LPS-induced lower respiratory inflammation. The primary outcomes included a marked reduction in peribronchiolar inflammation (Figure 6A) and a notable decrease in the migration of alveolar macrophage into the alveoli (Figure 6B), which are hallmarks of inflammation in this model. Notably, gross examination of internal organs, including the kidneys, heart, spleen, and liver, showed no pathogenic lesions, suggesting that KA1-P1 treatment did not cause systemic toxicity or damage.

**Figure 7:**
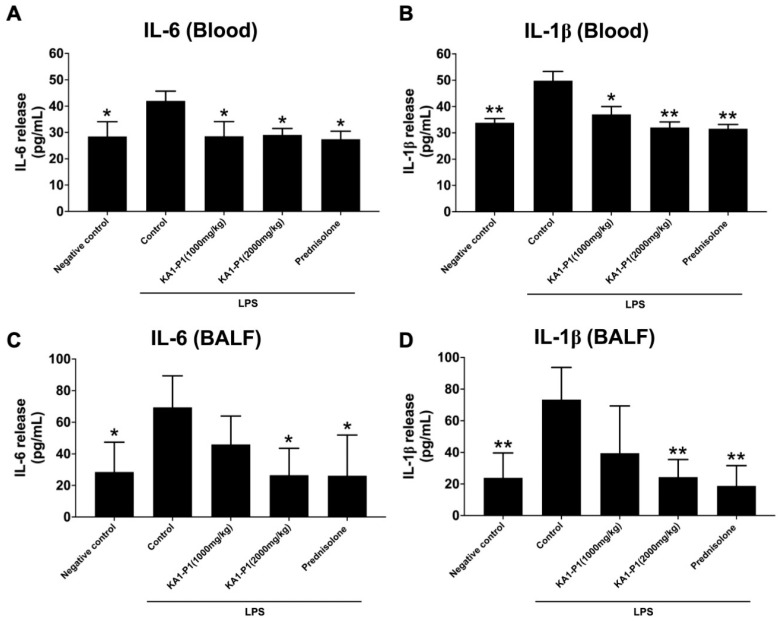
ELISA bronchoalveolar lavage fluid (BALF) and blood-based inflammatory cytokines (IL-6, IL-1β). C57BL/6NJcl mice (*n* = 40) were randomly divided into five groups (*n* = 8 for each group containing an equal number of males and females). The levels of IL-6 (**A**) and IL-1β (**B**) in blood and the levels of IL-6 (**C**) and IL-1β (**D**) in bronchoalveolar lavage fluid were measured using ELISA. Data are mean ± S.D. from at least in triplicate experiments; * *p* < 0.05 and ** *p* < 0.01 indicate statistically significant differences compared with the LPS + ATP-induced control group.

Furthermore, inflammatory cytokine levels (IL-6 and IL-1β) in plasma and bronchoalveolar lavage fluid (BALF) were measured and compared across different treatment groups over the 12-week period. The results showed a significant increase in IL-6 and IL-1β levels in the LPS-induced group compared to the normal control group in both blood plasma (*p* < 0.05) and BALF (*p* < 0.01). However, treatment with KA1-P1 (2000 mg/kg) significantly reduced IL-6 and IL-1β levels compared to the untreated group in both blood plasma (*p* < 0.05) and BALF (*p* < 0.01), as shown in Figure 7. A significant decrease in IL-6 and IL-1β levels was also observed in the low-dose KA1-P1 treatment group (1000 mg/kg), but only in blood plasma. While a reduction tendency was noted in BALF, the difference was not statistically significant. These findings suggest that KA1-P1 effectively attenuates LPS-induced lower respiratory inflammation in C57BL/6NJcl mice, as evidenced by reduced lung inflammation, decreased inflammatory cytokine levels (IL-6 and IL-1β) in plasma and BALF, and fewer inflammatory lung lesions observed in histopathological analysis.

## 4. Discussion

Chronic inflammation in aging serves as a double-edged sword. While it plays a beneficial role in early life by neutralizing harmful pathogens, it becomes detrimental later in life, leading to immune dysfunction and increased autoreactivity [41,42]. The activation of the NLRP3 inflammasome pathway in response to infectious pathogens is a key contributor to immunopathology, often resulting in prolonged inflammation and adverse health outcomes, particularly in older adults following viral or bacterial respiratory infection [1,5]. Given these concerns, this study focused on identifying anti-inflammatory strategies to mitigate chronic inflammation by investigating KA1-P1, an anthocyanin-rich fraction derived from the germ and bran of Kum Akha 1 CMU black rice.

Pigmented rice varieties, such as black and red rice, exhibit diverse nutritional profiles, with traditional landrace varieties often possessing superior nutraceutical properties compared to commercial cultivars [23,24,36]. In this study, we extracted and characterized the anthocyanin-rich fraction KA1-P1 from Kum Akha 1 CMU black rice using our previously described solvent-partitioning technique [28]. The total anthocyanin content of KA1-P1 (74.63 ± 1.66 mg/g extract) was comparable to values reported in previous studies [18,24]. HPLC analysis confirmed the presence of C3G and P3G as the major anthocyanin compounds, with C3G (45.58 ± 0.48 mg/g extract) and P3G (6.92 ± 0.29 mg/g extract) levels being consistent with earlier findings [24,43,44]. Prior studies have established a strong correlation between anthocyanin pigment content and antioxidant capacity in the pigmented rice [18,45]. Consistent with these findings, KA1-P1 demonstrated potent antioxidant activity, as confirmed by DPPH and ABTS assays. It could be inferred that C3G and P3G were the active ingredients of KA1-P1 responsible for the LPS + ATP-induced lung inflammation in our study. Previously, our study identified C3G and P3G as the active compounds of black rice germ and bran extracts, as they could exhibited anti-inflammatory properties against SARS-CoV-2 spike protein-induced A549 lung cells inflammation and THP-1 macrophages via inhibiting of NLRP3 inflammasome machinery proteins [24]. Similarly, the anti-inflammatory effect of KA1-P1 against LPS-ATP-induced A549 lung cells was potentially due to the presence of these two anthocyanins (C3G and P3G) in KA1-P1.

Several pigmented rice varieties have been investigated for their anti-inflammatory properties, with promising results. For example, anthocyanin-rich extracts from Kum Doi Saket black rice have been shown to suppress inflammation in LPS-induced RAW 264.7 macrophages by downregulating NF-kB and AP-1 signaling pathways, thereby reducing IL-6 and TNF-α secretion [28]. Additionally, extracts from Kum Doi Saket black rice germ and bran inhibited IL-6, IL-1β, and IL-18 production via NLRP3 inflammasome suppression in SARS-CoV2-spike protein-induced A549 lung and THP-1 cell lines [24]. Similarly, proanthocyanidin-rich fractions from Yamuechaebia 3 red rice effectively reduced LPS + ATP-induced lung inflammation by inhibiting the NF-kB/NLRP3 inflammasome pathway [36]. Building on these findings, our study investigated the anti-inflammatory effects of KA1-P1 on LPS + ATP-induced A549 lung epithelial cells by evaluating its impact NLRP3 inflammasome-related genes and proteins. Unlike previous studies limited to in vitro models, we extended our investigation to an in vivo C57BL/6NJcl mice model to evaluate the efficacy of KA1-P1 against lower respiratory tract inflammation.

Exposure to pathogens can activate lung inflammation through the NLRP3 inflammasome pathway, leading to the release of IL-1β and IL-18. These cytokines further stimulate IL-6 and TNF-α, amplifying the inflammatory cascade and contributing to chronic inflammation and lung tissue damage [46,47]. Additionally, NLRP3 activation enhances TGF-β1 secretion, promoting collagen deposition and pulmonary fibrosis [48,49]. Given its pivotal role in bacterial or viral pneumonia, targeting the NLRP3 inflammasome presents a promising therapeutic strategy. To mimic lung inflammation in vitro, we employed LPS as a priming agent (pathogen-associated molecular pattern, PAMP) and ATP as a secondary activator (damage-associated molecular pattern, DAMP), which, together, fully activate the NLRP3 inflammasome and trigger inflammatory responses in lung epithelial cells inflammation [50,51]. This model has been widely used in previous studies [52,53]. Our results demonstrated that KA1-P1 significantly suppressed the expression of IL-6, IL-1β, and IL-18 at both gene and protein levels in LPS + ATP-induced A549 cells. This suggests that KA1-P1 effectively inhibits inflammation via the downregulation of the NLRP3 inflammasome signaling pathway.

Consistent with previous reports, anthocyanin-rich butanol fractions from black rice extracts exhibited potent anti-inflammatory effects in RAW264.7 and THP-1 macrophages by inhibiting cytokine production, whereas water fractions showed no such effects [24,28,54]. Our findings align with these observations, as the anthocyanins were concentrated in KA1-P1 (butanol fraction) rather than KA1-P2 (water fraction), which also corresponded to its higher antioxidant capacity. This highlights the significance of anthocyanins, particularly C3G and P3G, as key contributors to the anti-inflammatory potential of KA1-P1. The NLRP3 inflammasome pathway has been implicated in various lower respiratory diseases, including influenza, tuberculosis, bacterial pneumonia, and streptococcus pneumoniae infections, particularly in older adults [55,56,57]. Even after pathogen clearance, persistent bacterial particles (PAMPs) and cellular debris (DAMPs) can contribute to stimulating inflammation via Toll-like receptors (TLRs) and nucleotide-binding oligomerization domain-like receptors (NLRs), leading to chronic inflammatory states. The activation of NLRP3 inflammasome machinery, including NLRP3, ASC, and caspase-1, results in IL-1β and IL-18 secretion, further propagating the inflammatory response [51,58].

It can be hypothesized that the inhibition of KA1-P1 on the lung cells’ inflammation is not only achieved by regulating the NLRP3 inflammasome pathway, but also other upstream regulatory pathways such as CAMK2A oxidative stress, ER stress–CHOP pathway, NF-κB, MAPK, and JAK/STAT pathways. Briefly, the upregulation of CAMK2A, a pathological mediator of oxidative stress, may be driven by PAMPs or DAMPs, triggering immune responses, leading to the production of pro-inflammatory cytokines and activating NLRP3 inflammasome signaling [59]. Additionally, LPS has also been reported to induce inflammation through the ER stress-induced calcium–CHOP pathway in lung epithelial cells [60]. The NLRP3 inflammasome pathway has been implicated in various respiratory diseases. At the molecular level, the presence of bacteria or cellular stress led to the generation of PAMPs or DAMPs. These molecules stimulate inflammation by signaling through TLR or NLR and then activating the NF-κB- [61], AP-1- [62], or JAK/STAT-related signaling pathways [63]. Further studies on the identification of candidate upstream pathways that are also regulated by KA1-P1 could offer the potential targeted therapy on the precise inhibition of NLRP3 inflammasome-driven lung inflammation.

NLRP3-induced inflammation and the release of IL-1β and IL-18 are linked to various respiratory diseases. While existing drugs, such as canakinumab, anakinra, and rilonacept, block IL-1β, NLRP3 activation also produces IL-18 and other cytokines, potentially limiting the effectiveness of IL-1β inhibitors and causing unintended immunosuppressive effects. Thus, targeting the NLRP3 inflammasome directly may offer a better therapeutic approach [64]. Natural compounds like flavonoids and anthocyanins possess anti-inflammatory properties, with some found to inhibit NLRP3 inflammasome assembly or caspase-1 activity, while others act as downregulators of inflammasome components, reducing caspase-1 activation and cytokine production [65]. In this study, KA1-P1, rich in anthocyanins, was found to suppress lung inflammation by inhibiting NLRP3 inflammasome proteins and cytokine releases. However, the precise mechanism—whether through direct cytokine inhibition or the downregulation of inflammasome components—remains unclear. Further mechanistic studies are needed to better understand KA1-P1’s anti-inflammatory effects and its potential as an alternative therapy for NLRP3-related inflammatory diseases in the lungs and other organs.

Our in vivo study investigated the effects of KA1-P1 in LPS + ATP-induced C57BL/6NJcl mice. Both high- and low-dose KA1-P1 treatments reduced peribronchiolar inflammation and alveolar macrophage infiltration, which are key indicators of pulmonary inflammation. These findings suggest that KA1-P1 modulates inflammatory responses in the lung, thereby mitigating tissue damage associated with LPS-induced injury. A critical aspect of this study was the assessment of inflammatory cytokines, specifically IL-6 and IL-1β, which are well-established mediators of acute and chronic inflammation in respiratory diseases. Regarding the inflammatory markers for LPS + ATP-induced lung inflammation in our study, IL-6 represented the cytokine that play an active role in mediating a number of inflammatory lung diseases. IL-6 enhances immune cell recruitment and promotes tumor progression by inducing epithelial-to-mesenchymal transition (EMT) and angiogenesis [66], while IL-1β and IL-18 are key pro-inflammatory cytokines activated by the NLRP3 inflammasome pathway. IL-1β promotes neutrophilic inflammation, epithelial cell damage, and fibrosis formation, and IL-18 contributes to immune cell activation and activating the release of other cytokines. Their dysregulation is linked to chronic inflammatory lung diseases [49].

As expected, LPS exposure led to a significant increase in IL-6 and IL-1β levels in both blood plasma and BALF. However, treatment with KA1-P1 (2000 mg/kg) markedly reduced the levels of these cytokines, suggesting a strong anti-inflammatory effect. Interestingly, the low dose KA1-P1 treatment (1000 mg/kg) significantly decreased IL-6 and IL-1β levels in plasma but only showed a non-significant trend of reduction in BALF. This dose-dependent effect indicates that higher doses of KA1-P1 may be more effective in suppressing pulmonary inflammation, while lower doses might primarily exert systemic anti-inflammatory effects. These findings align with previous studies on bioactive compounds with anti-inflammatory properties, which have demonstrated their ability to downregulate inflammatory cytokines and inhibit immune cell infiltration in lung tissue. The observed reduction in alveolar macrophage migration further supports the potential of KA1-P1 in modulating immune responses in the lung microenvironment. Aging-related immune dysfunction often results in chronic activation of the NLRP3 inflammasome, contributing to lung inflammation, ARDS, and fibrosis [67,68]. The accumulation of inflammatory mediators, such as IL-1β, TNF-α, and interferons, can accelerate lung tissue damage [69]. Prior studies have demonstrated that C3G from black rice suppressed LPS-induced inflammation in RAW 264.7 macrophages and carrageenan-induced inflammation in air pouches in BALB/c mice through the suppression of the pro-inflammatory cytokines, TNF-α and IL-1β, mechanistically via regulating NF-κB and MAPK activation [25,26,28].

In this study, the selected doses of KA1-P1 for the animal model were based on existing toxicological data for anthocyanins, particularly their acute and sub-chronic toxicity profiles. Previous reports have demonstrated that anthocyanins extracted from currants, blueberries, and elderberries exhibit no toxic effects at doses exceeding 2000 mg/kg body weight (BW) in both rats and mice (LD_50_ > 2000 mg/kg BW). Additionally, sub-chronic toxicity studies have shown that rodents administered anthocyanins at doses of 125–500 mg/kg BW daily for six months exhibited no mortality or adverse effects [70]. Given that KA1-P1, in our study, contains 74.63 mg of total anthocyanins per gram of extract, the selected doses of 1000 mg/kg and 2000 mg/kg KA1-P1 correspond to approximately 75 mg/kg and 150 mg/kg total anthocyanins, respectively. These concentrations have demonstrated biological activity in mice models while remaining within the safe range established by previous toxicological studies [71].

The typical dietary intake of anthocyanins in humans varies, with estimated exposure through a regular diet reaching approximately 1 mg/kg BW per day for adults and 2 mg/kg BW per day for children [72]. In comparison of other studies in human subjects, a clinical trial reported that 320 mg daily anthocyanin supplementation significantly decreased NLRP3 inflammasome-related markers (caspase-1, IL-1β, and IL-18) in PBMCs and plasma of NAFLD patients [73]. The 300 mg anthocyanins/day as a black rice germ and bran instant powder was sufficient to promote the immunomodulatory effects in human subjects as well as reduced inflammatory cytokine levels (IL-6 and *C*-reactive protein) in aging population [27]. The information regarding the anti-inflammation dosages of KA1-P1 in this study could improve the study’s applicability to functional food recommendations for the further use of black rice germ and bran in clinical settings.

Anthocyanins from black rice germ and bran extracts undergo bioactivation and partial degradation due to β-glucosidase activity from microbiota and salivary enzymes. However, glycoside forms like peonidin (P3G) and cyanidin (C3G) remain stable in the oral cavity. In the stomach, anthocyanins exhibit high stability in acidic conditions and are absorbed into systemic circulation via bilitranslocase-mediated transport [74]. Studies show that 68–80% of anthocyanins appear in human urine as metabolites, indicating rapid metabolism and elimination [75]. The pharmacokinetics of anthocyanins are complex, with metabolite peaks between 2 and 30 h post-consumption and elimination half-lives ranging from 0.5 to 96 h, influenced by enterohepatic recirculation and microbial metabolism [76]. Regarding the pharmacokinetic in animals and human studies, a 2009 study in mice showed a shorter half-life of C3G (0.7–1.8 h) following a 500 mg/kg oral dose [77]. A 2013 study on humans found that after a 500 mg oral dose, C3G metabolites had half-lives between 12.44 and 51.62 h, suggesting prolonged presence in the blood circulation [78]. Furthermore, anthocyanins were also detected in the lung tissue following a bolus dose (10 mg/animal) by oral gavage in blueberry-fed mice. On the other hand, when the tissue anthocyanins were converted to their respective aglycons by acid hydrolysis and selectively extracted in glycoside, forms including C3G and P3G were also detected in the lung tissue, indicating that anthocyanins can reach and exert their effects beyond the GI tract [79]. While P3G’s pharmacokinetics in humans remain unclear, its similarity to C3G suggests comparable metabolism. Further studies are needed to determine its precise bioavailability and therapeutic potential.

Interestingly, it is worthwhile to highlight the importance of anti-inflammatory effects of KA1-P1 in the context of controlling the inflammation through the gut–lung axis. The gut–lung axis highlights the interplay between gut microbiota and respiratory health, influencing immune response and airway respiratory homeostasis [80]. Although gut and lung microbiota differ in composition, they share similarities in epithelial origins (endoderm), anatomical structure, and early microbial colonization. Regulating the gut microbiota has been linked to reducing respiratory infections [81]. It can be hypothesized that the anti-inflammatory effects of KA1-P1 are partly due to immune modulation through the gut–lung axis. Anthocyanins are efficiently absorbed in the small intestine, undergoing metabolism into bioactive derivative forms. Gut microbiota (Lactobacillus and Bifidobacterium) enhance anthocyanin bioavailability by breaking them down into phenolic acids with strong anti-inflammatory and antioxidant properties [82]. Additionally, anthocyanins promote beneficial gut bacteria while inhibiting harmful strains, contributing to microbial balance. Moreover, the NLRP3 inflammasome is a key immune regulator in both the gut and lungs, mediating inflammatory cell recruitment. While gut immunity clears most bacterial infections, some escape into circulation, reaching the lungs and triggering inflammation. NLRP3 activation leads to caspase-1 activation and the release of IL-1β and IL-18 pro-inflammatory cytokines which are associated with airway inflammation in COPD [83]. Therefore, future study targeting the gut microbiota may offer a potential strategy for managing NLRP3 inflammation-related respiratory diseases.

Therefore, the in vivo anti-inflammatory effects of KA1-P1 against LPS-induced pulmonary inflammation, similar to in vitro findings, could potentially be the result of the presence of C3G and P3G anthocyanins in KA1-P1. Nevertheless, further study on the single or combined administration of these anthocyanins to LPS-induced mice could offer more understanding towards their inhibitory mechanism of these active ingredients from KA1-P1. In this study, we investigated inflammation in the lower respiratory tract, selecting the lung as the final site of inflammatory progression. To assess cytokine levels (IL-6 and IL-1β), we analyzed bronchoalveolar lavage fluid (BALF) and blood, as BALF provides a well-established method for evaluating airway and alveolar inflammation [84]. While bronchi-specific experimentation was not conducted, the inflammatory response in the broncho–pulmonary system is reflected in BALF cytokine levels. Further studies focusing on bronchial tissue-specific analysis may provide additional mechanistic insights into airway inflammation inhibition of anthocyanins from black rice germ and bran. Importantly, KA1-P1 treatment did not result in any pathological changes in internal organs, including the kidneys, heart, spleen, and liver, indicating its safety profile over the 12-weeks treatment period. Therefore, it would be of interest to investigate whether KA1-P1 also affects systemic inflammatory makers beyond the lungs. Further study on measuring cytokine levels (IL-6, TNF-α, and IL-1β) in other tissues, such as the liver or spleen, could provide a broader perspective on its anti-inflammatory effects. This suggests that KA1-P1 may be a promising candidate for further investigation as a therapeutic agent for inflammatory lung diseases, including ARDS and COPD, which are often associated with excessive cytokine release and immune cell infiltration.

Regarding the in vivo model use in our study, the LPS-induced lung inflammation model in mice is widely used to study human respiratory diseases such as ARDS and COPD [85,86]. It effectively mimics key inflammatory responses, including cytokine release and immune cell recruitment that can be detected by blood levels and bronchoalveolar fluid as well as the histopathology of respiratory tissues. Previous studies found that environmental or occupational exposure to inhaled LPS has been associated with the development of COPD [87]. Briefly, exposure to LPS causes reversible inflammation of airways, lung parenchyma architecture changes associated with airflow obstruction, and airway remodeling in mice that results in emphysema. However, species-specific differences in immune response should be considered when translating findings to humans. Variations in innate immune signaling, cytokine profiles, and lung architecture between animals and humans may influence disease progression and treatment efficacy [88]. Understanding these differences can help refine pre-clinical models and improve the translational research findings for therapeutic development.

Taken together, the protective effects of anthocyanins obtained from black rice germ and bran can be utilized in potential preventive strategies that incorporate pigmented rice as a functional food product. Moreover, anthocyanin-rich extracts from black rice germ and bran can be formulated into nutraceutical products, such as supplements or beverages, which could be used as dietary interventions for target populations, including aging individuals and vulnerable people suffering from chronic systemic illnesses such as diabetes, cardiovascular diseases, and inflammation-related long COVID, to improve quality of life and overall health.

## 5. Conclusions

Overall, our findings highlight that the anthocyanin-rich fraction KA1-P1 from black rice germ and bran exerts strong anti-inflammatory effects, both in vitro and in vivo, by suppressing the NLRP3 inflammasome pathway. These results support the therapeutic potential of KA1-P1 for treating chronic inflammatory diseases associated with NLRP3 activation, including bacterial and viral pneumonia, ARDS, and age-related lung inflammation. Although C57BL/6NJcl mice are widely used in inflammatory research, they may not fully represent the complexity of human immune responses. Species-specific differences in immune function could impact the translational relevance of the results. Further investigations, including clinical trials, are warranted to explore its efficacy in human populations.

## Figures and Tables

**Table 1:** The primers for inflammatory genes used for qRT-PCR analysis [36].

Gene	Sequences
NLRP3	Forward, 5′-AAC ATG CCC AAG GAG GAA GA-3′
	Reverse, 5′-GGC TGT TCA CCA ATC CAT GA-3′
IL-1β	Forward, 5′-TGC TCA AGT GTC TGA AGC AG-3′
	Reverse, 5′-TGG TGG TCG GAG ATT CGT AG-3′
IL-18	Forward, 5′-TCG GGA AGA GGA AAG GAA CC-3′
	Reverse, 5′-TTC TAC TGG TTC AGC AGC CA-3′
IL-6	Forward: 5′-ATG AAC TCC TTC ACA AGC-3′
	Reverse: 5′-GTT TTC TGC CAG TGC CTC TTT G-3′
GAPDH	Forward, 5′-TCA ACA GCG ACA CCC AC-3′
	Reverse, 5′-GGG TCT CTC TCT TCC TCT TGT G-3′

**Table 2:** Phytochemical characteristics of KA1 black rice germ and bran extracts (KA1-P1 and KA1-P2).

Phytochemicals	KA1-P1	KA1-P2
Total phenolic contents (mg GAE/g extract)	168.82 ± 7.65 **	73.62 ± 0.69
Total flavonoid contents (mg CE/g extract)	97.45 ± 6.96 **	39.29 ± 4.11
Total anthocyanins (mg/g extract)	74.63 ± 1.66 **	33.26 ± 1.11
Cyanidin-3-glucoside (C3G) (mg/g extract)	45.58 ± 0.48	ND
Peonidin-3-glucoside (P3G) (mg/g extract)	6.92 ± 0.29	ND

Data are presented as mean ± S.D. values of at least in triplicate. GAE; gallic acid equivalent, CE; catechin equivalent, ND; not detected ** *p* < 0.01 indicate statistically significant differences compared with KA1-P2 for respective phytochemical tests.

**Table 3:** Antioxidant activities of KA1 black rice germ and bran extract.

Extracts	IC_50_ (μg/mL)	
	DPPH Assay	ABTS Assay
KA1-P1	47.82 ± 4.65 *	17.35 ± 0.66 *
KA1-P2	215.10 ± 28.23	60.05 ± 0.42
Vitamin E	24.74 ± 0.60	-
Trolox	-	2.45 ± 0.23

Data are presented as mean ± S.D. values of at least in triplicate. * *p* < 0.05 indicate statistically significant differences compared with KA1-P2 for respective antioxidant tests.

## Data Availability

Data are contained within the article.
